# Frontalis Muscle Flap Suspension for the Correction of Congenital Blepharoptosis in Early Age Children

**DOI:** 10.1371/journal.pone.0053185

**Published:** 2013-01-07

**Authors:** Dianju Hou, Gehong Li, Lin Fang, Bing Li

**Affiliations:** 1 Department of Microinvasive Plasitic Surgery Center, Plastic Surgical Hospital, Peking Union Medical College and Chinese Academy of Medcial Sciences, Beijing, P.R. China; 2 Department of Hospital Infection-Control, Plastic Surgical Hospital, Peking Union Medical College and Chinese Academy of Medcial Sciences, Beijing, P.R. China; The University of Tennessee Health Science Center, United States of America

## Abstract

**Background:**

We aimed to report our successful use of frontalis muscle flap suspension for the correction of congenital blepharoptosis in early age children.

**Methods:**

This retrospective study included 61 early age children (41 boys, 20 girls) with an average age of 6 years (range, 3–10 years) with congenital blepharoptosis who received surgery during the period from March 2007 to January 2011. There were 39 cases of unilateral blepharoptosis and 22 cases of bilateral blepharoptosis, thus a total of 83 eyes were affected. If patient had bilateral blepharoptosis, both eyes were operated on in the same surgery. Patients were followed for 3 months to 5 years. The procedure was performed without complications in all cases.

**Results:**

The postoperative healing grade was good in 81 eyes (97.6%); the correction of blepharoptosis was satisfactory, the double eyelid folds were natural and aesthetic, the eyelid position and the curvature were ideal, and the eyes were bilaterally symmetrical. The postoperative healing grade was fair in 2 eyes (2.4%); blepharoptosis was improved compared with that before surgery. At discharge, lagophthalmos was noted in 10 eyes of which 4 cases resolved by the last follow-up. The remaining 6 cases were mild. Eleven eyes received reoperation for residual ptosis after the first surgery. The curvature of the palpebral margin was not natural in 4 eyes. These unnatural curvature possibly was caused by an excessively low lateral fixation point or postoperative avulsion.

**Conclusion:**

Frontalis muscle flap suspension under general anesthesia for the correction of congenital blepharoptosis in early age children can achieve good surgical results.

## Introduction

Children with congenital blepharoptosis (abnormal low-lying upper eyelid margin with the eye in primary gaze) may gradually develop a habit of extending the head, frowning, and shrugging while looking at objects because their view is obstructed, which may affect the normal development of the cervical spine and cause *amblyopia ab non usu* (disuse amblyopia), myopia, and astigmatism [1]. These conditions can seriously impact on the physical and psychological development of children. In the majority of cases, surgical correction is needed; however, consensus regarding the most appropriate age to perform surgery is lacking [1]–[4]. Some authors consider 4–5 years of age the most appropriate because postoperative lagophthalmos (the inability to close the eye completely) is tolerated better than if the surgery is performed earlier, while other authors have reported good results in children <3 year of age [2], [5].

Regardless of age, surgical correction of congenital blepharoptosis in children is challenging as the tissue of the eyelids and surrounding area is vulnerable, coping with surgery is hard, and it is difficult to position the eyes under general anesthesia [3]. Many techniques for the correction of congenital blepharoptosis using autologous fascia lata and permanent suture material have been described, and the choice of technique depends on many factors including the age of the patient and the severity of ptosis [1], [3], [6], [7]. The most commonly used technique is frontalis muscle suspension, and various modifications have been described [4], [8]–[12].

The purpose of this study is to report our successful use of frontalis muscle flap suspension for the correction of congenital blepharoptosis in early age children under general anesthesia.

## Patients and Methods

This retrospective study included 61 early age children (41 boys, 20 girls) with an average age of 6 years (range, 3–10 years) with congenital blepharoptosis who received surgery during the period from March 2007 to January 2011. All diseased eyes were diagnosed as congenital blepharoptosis, except for three eyes in patients with bilateral blepharoptosis which were due to microphthalmia (a developmental disorder in which one or both eyeballs are abnormally small). The majority of the blepharoptosis cases in this report were isolated or idiopathic. No other syndromic blepharoptosis was observed. If patient had bilateral blepharoptosis, both eyes were operated on in the same surgery. This study was approved by the Institutional Review Board (IRB) of the Plastic Surgical Hospital, Peking Union Medical College. The parents or guardians of all children provided written informed consent for plastic surgery treatment and provided verbal informed consent for the possible publication of surgical information and eye photos.

A complete family history and history of the disease including onset was performed for all patients to diagnosis congenital, acquired, or systemic ptosis. Detailed ophthalmic and orthoptic examinations were performed on all children. Preoperative routine examinations included observation of Bell phenomenon and the level of the upper eyelid margin, measurement of the retained function of the levator muscle and frontal muscle, vision screening, and general physical examination. For this surgery, measurement of the retained function of the levator muscle should be <7 mm, and the function of the frontal muscle should be normal. We also considered cosmetic, visual (where the lid occluded the visual axis), and the presence of a significant compensatory head posture in determining the surgical method.

**Figure 1:**
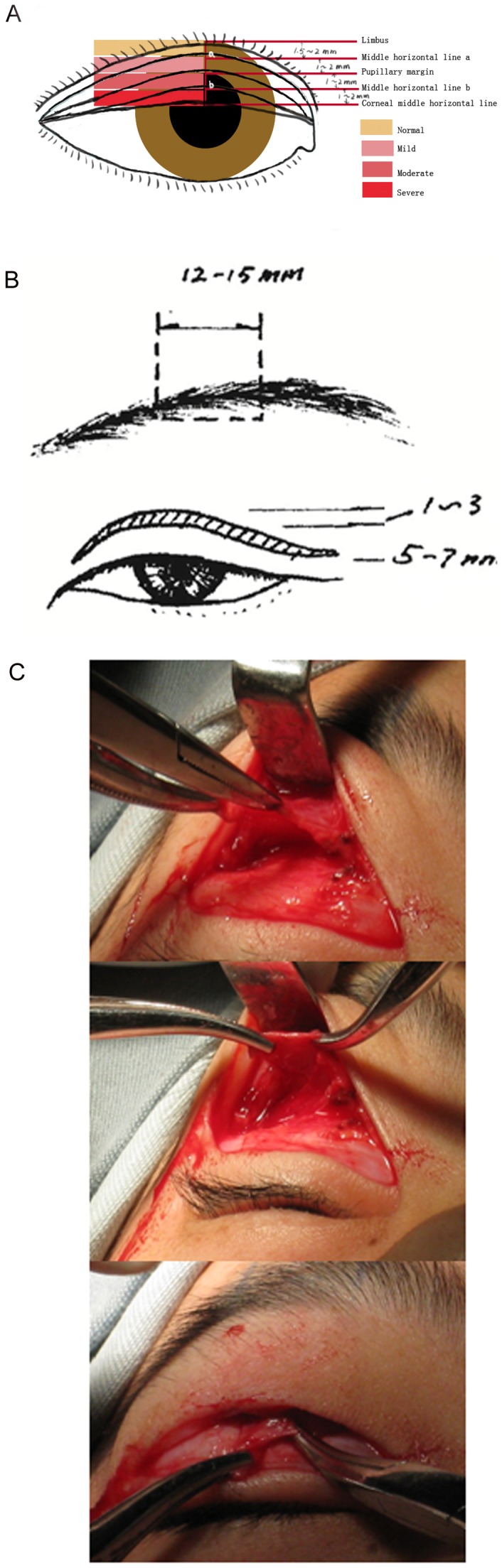
Schematic diagram of the curvature of the eyelid margin, surgical design and incision in blepharoptosis surgery. (A) Normal eyelid margin and varying degrees of ptosis with average measurements in a young child. The different color areas indicate the possible location of the eyelid margin in different ptosis severities. (B) The width of the frontal muscle flap is 12–15 mm, and the height of dissociation depends on the clinical circumstances. The resection of the affected upper eyelid skin was 1–3 mm and the width of the double eyelid fold is 5–7 mm. (C) Representative intraoperative photos.

According to the location of the eyelid, ptosis can be divided into mild, moderate, and severe (Fig. 1A). Mild ptosis is considered if the edge of the eyelid is above the pupil, drooping 1–2 mm; moderate ptosis is the eyelid covering the pupil, drooping 3–4 mm; severe ptosis is the edge of the eyelid at the central level of the pupil covering the 1/2 the area, drooping more than 4 mm. The majority of cases (about 90%) were severe or moderate ptosis before surgery.

### Surgical method

An illustration of the surgical design is shown in Fig. 1B and C. The incision for double eyelid plasty was used. After induction of general anesthesia with oral or nasal intubation, or administration of local anesthesia, methylene blue was used to mark the incision line based on the size of the palpebral fissure (separation between the upper and lower eyelids), the degree of blepharoptosis, and degree of slack of the upper eyelid skin. The widest point was 5–7 mm from the palpebral margin. Generally, no skin needed to be removed on the unaffected side. On the affected side, 1–3 mm skin was removed according to the degree of slack in the skin.

The face was disinfected with 75% ethanol and sterile drapes were placed. The surgical area was injected with a mixture of 0.25% lidocaine and 1/200,000 epinephrine. The skin and subcutaneous tissue was incised along the incision line, and part of the pre-tarsal orbicularis muscle was resected to expose the upper edge of the tarsus. If an incision under the eyebrow was used, the skin, subcutaneous tissue, and muscle layer was cut open along the incision line to expose the frontalis muscle flap. If no incision under the eyebrow was made, the orbital septum was cut open, and blunt dissection was performed upward behind the orbital septum until the lower edge of the eyebrow. The decision to use an incision under the eyebrow was based on the surgeon's preference. An L-shaped retractor was used to create and elevate a tunnel, and a No. 11 scalpel was used to incise the tissue from the muscle layer to the subcutaneous layer at the lower edge of the eyebrow and to dissect upward until above the eyebrow exposing the frontalis muscle flap. A 3-0 silk thread was placed for downward traction.

Ophthalmic scissors were used to dissect upward subcutaneously and below the frontalis muscle according to the degree of dissociation of the frontalis muscle flap. The bilateral frontal incisions should not be too long, especially the lateral incision: an appropriate length allows adequate traction of the frontalis muscle flap without causing skin adhesions or eyebrow displacement. The degree of the dissociation of the frontalis muscle flap and the skin should not be excessively great because such a range may cause excessive injury and form an excessive long frontalis muscle flap. This type of unwanted flap can cause an insufficient suspension force and alter the desirable effects on eyelid motility.

The dissociation on the medial side did not exceed the supraorbital notch, on the lateral side did not exceed the eyebrow peak, and the width was 12–15 mm. The range of dissociation above the eyebrow was determined according to the dissociation of the frontalis muscle flap. Dissociation was performed at two levels, subcutaneously and below the frontalis muscle in the eyebrow area and the area above the eyebrow. Dissociation below the orbicularis muscle or behind the orbital septum was performed in the upper eyelid.

After hemostasis was assured, the frontalis muscle flap was pulled downward until the upper edge of the tarsus, and it was sutured at the midpoint of the tarsus and the medial and lateral 1/3 of the tarsus using a horizontal mattress suture. The appropriate height of suspension was to lift the midpoint of the affected upper palpebral margin upward until it was 1.5 mm or more above the upper edge of the iris, and to make the height of the lateral fixation point slightly higher than that of the midpoint. Attention was paid to the height of the bilateral fixation points and the midpoint so that a natural curvature of the palpebral margin was formed. After assuring hemostasis, 7-0 nylon suture was used to suture the skin incision as in double eyelid plasty. A drainage strip was placed if a large amount of oozing blood was noted. Erythromycin eye ointment was applied in the palpebral fissure to prevent infection, and a pressure dressing was placed on the frontal and eyebrow areas. The sutures were removed under general or local anesthesia 5–7 days after surgery.

### Outcome measures

Patients were followed for 3 months to 5 years, with a median follow-up of 6 months. Outcome measures included correction of blepharoptosis, healing, complications, and lagophthalmos. Lagophthalmos is generally divided into 3 degrees. Mild lagophthalmos is the exposure of only a small amount of conjunctive. The eyes can be forcibly closed, but remain open during sleep. In moderate lagophthalmos the eye cannot be closed but the cornea is not exposed. In severe lagophthalmos the eyes cannot be closed.

The healing was graded as follows: Good, the eyelid is slightly swollen with some numbness, without discoloration or bruising; Fair, the eyelid is swollen and feels numb, with mild discoloration or bruising; and Poor, the presence of complications such as infection or severe lagophthalmos and ultimately conspicuous scaring. Satisfactory correction of blepharoptosis was defined as: 1) Normal upper eyelid function; 2) The eyelid fold, position, and curvature are natural and aesthetic, and the bilateral eyes are symmetrical; and 3) No postoperative short-term or long-term complications such as severe lagophthalmus.

## Results

There were 39 cases of unilateral blepharoptosis and 22 cases of bilateral blepharoptosis, thus a total of 83 eyes were affected and received surgery (Table 1). All diseased eyes were diagnosed as congenital blepharoptosis except for 3 eyes in patients with bilateral blepharoptosis, which were due to microphthalmia. The surgical time, anesthesia method, blood loss, healing status, hypophasis at discharge, follow-up time, and need for reoperation are summarized in Table 1. Data of each individual case are presented in Table S1. The majority eyes had moderate (23 out of 83) or severe (52 out of 83) preoperative ptosis. The procedure was performed without complications in all cases. The average surgical time for bilateral eyes was longer than that for unilateral eyes (65 min vs. 50 min). Only 11 eyes received the local anesthesia method, while other 72 eyes received general anesthesia for the surgeries. The postoperative healing grade was good in 81 eyes (97.6%); the correction of blepharoptosis was satisfactory, the double eyelid folds were natural and aesthetic, the eyelid position and the curvature were ideal, and the eyes were bilaterally symmetrical. The postoperative healing grade was fair in 2 eyes (2.4%); blepharoptosis was improved compared with that before surgery.

**Table 1:** Pre- and postoperative patient data.

	Unilateral Eyes	Bilateral Eyes
Number of patients	39 (R17/L22)	22 (R22/L22)
Age (years)	6 (3–9)	6 (3–10)
Male	26 (66.7)	15(68.2)
Diagnosis		
Congenital blepharoptosis	39 (100)	44 (100)
Microphthalmia	0	3 (6.8)
Preoperative ptosis		
Mild	4 (10.3)	2 (9.1)
Moderate	13 (33.3)	5 (22.7)
Severe	22 (56.4)	15 (68.2)
Surgical time (min)	50 (35–140)	65 (35–125)
Follow-up (months)	6 (3–12)	6 (3–60)
Number of hospital admissions		
1	34 (87.2)	18 (81.8)
2	4 (10.3)	3 (13.6)
3	1 (2.6)	1 (4.5)
Anesthesia		
Local	7 (17.9)	2 (9.1)
General	32 (82.1)	20 (90.9)
Healing grade		
Good	39 (100)	42 (95.5)
Fair	0 (0)	2 (4.5)
Blood loss (ml)		
<5	35 (89.7)	36 (81.8)
>5	4 (10.3)	8 (18.2)
Lagophthalmos at discharge, eyes	8 (25.5)	2 (4.5)
Lagophthalmos at last follow-up, eyes	4 (10.3)	2 (4.5)
Reoperation for residual ptosis, eyes	3 (7.7)	8 (18.2)

Data are presented as number (%) or median (range).

At discharge, lagophthalmos was noted in 10 eyes of which 4 cases resolved by the last follow-up. The remaining 6 cases were mild. Three eyes in unilateral eye group and 8 eyes in bilateral group received reoperation for residual ptosis after the first surgery. The curvature of the palpebral margin appeared unnatural in 4 eyes. This unnatural curvatures were possibly caused by an excessively low lateral fixation point or postoperative avulsion. There were no other complications or anomalies associated with the surgical technique.

**Figure 2:**
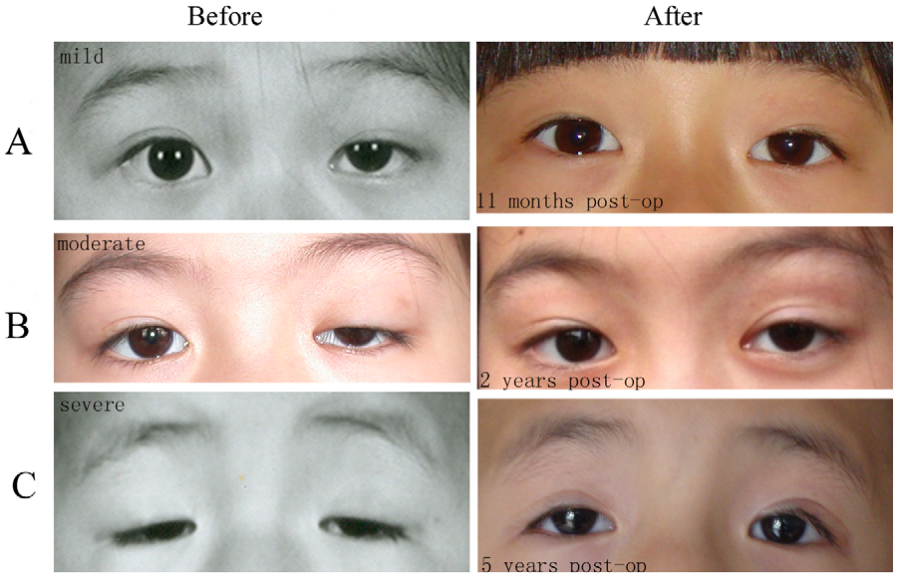
Representative pre- and postoperative photos of patients. (A) Mild ptosis of the left eye. (B) Moderate ptosis of the left eye. (C) Severe ptosis of both eyes. Satisfactory operative outcomes were observed at 11 months (A), 2 years (B), and 5 years (C).

Representative photos for mild ptosis (Fig. 2A), moderate ptosis (Fig. 2B) and severe ptosis (Fig. 2C) before and after the surgery are shown. According to the follow-up at 11 months, 2 years and 5 years after the surgery, the outcomes of the surgery were satisfactory.

## Discussion

In this report of the surgical correction of congenital blepharoptosis in early age children using a modification of frontalis muscle flap suspension, in 81 of 83 eyes (97.6%) the correction of blepharoptosis was satisfactory, the double eyelid folds were natural and aesthetic, the eyelid position and the curvature were ideal, and the eyes were bilaterally symmetrical. There were no surgical complications, 6 cases of lagophthalmos at last follow-up, and 8 eyes required re-operation for residual ptosis. The possible reasons for the failure were damage to the frontalis muscle flap during its preparation, weakness of the frontalis muscle, or the suspension height of frontalis muscle flap was not high enough.

**Figure 3:**
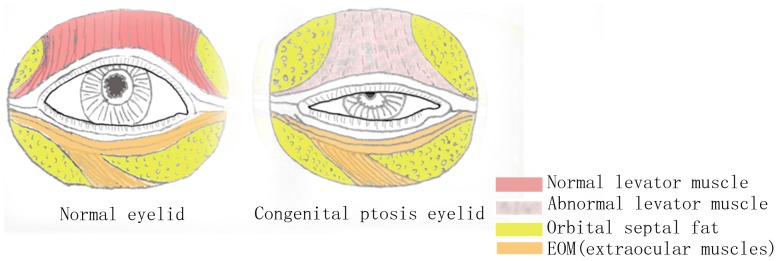
Illustration of a normal eyelid (left) and one with congenital blepharoptosis (right). In congenital blepharoptosis the levator palpebrae superioris, which elevates the upper eyelid, is damaged or weakened.

Blepharoptosis refers to partial or complete upper eyelid drooping caused by dysfunction or function loss of the levator palpebrae superioris (innervated by the oculomotor nerve; Fig. 3) and the Müller smooth muscle (innervated by the cervical sympathetic nerve) [13]. The upper eyelid may partially cover the pupil in mild cases, and completely cover the pupil in severe cases. This condition not only impairs appearance and vision, but also can cause severe amblyopia in patients with congenital blepharoptosis if left untreated [6]. In order to overcome the visual impairment, patients often tighten the frontalis muscle to elevate the position of the upper palpebral margin, resulting in deepened horizontal wrinkles of the frontal skin and upright eyebrow. Patients with bilateral blepharoptosis often develop a special gesture of head extending and frown since they need to extend the head to look at objects.

Generally, the cause of most cases of congenital blepharoptosis is idiopathic. Histologically, congenital blepharoptosis may be due to a myogenic, neurogenic, or aponeurotic cause. Congenital blepharoptosis can be isolated or syndromic. In our cases, all diseased eyes were diagnosed as congenital blepharoptosis except for 3 eyes in patients with bilateral blepharoptosis, which were due to microphthalmia. The most common cause of congenital ptosis is myogenic, due to the improper development of the levator muscle [14].

Correction of congenital blepharoptosis as early as possible has become increasingly necessary in modern society [7], [13]. There are many methods for the surgical correction of blepharoptosis, and 2 commonly used are levator palpebrae superioris shortening and frontalis muscle flap (or frontalis muscle myofascial flap) suspension [1], [3], [6]. Each method has its corresponding advantages, disadvantages, complications, and indications. Levator palpebrae superioris shortening is appropriate for mild to moderate blepharoptosis when the muscle strength of the levator palpebrae superioris >5 mm [1], [3], [6]. For moderate to severe cases when the muscle strength of the levator palpebrae superioris <4 mm, frontalis muscle flap (or frontalis muscle myofascial flap) suspension surgery is appropriate [1], [3], [6]. Various modifications of the commonly used techniques have been described. Frontalis suspension has been described using autogenous tendons [15], fascia lata strips [12], and autogenous temporal fascia [12]. Arajy et al. [16] described an open loop fascial sling for the treatment of severe congenital blepharoptosis and reported a satisfaction rate of 77% and found that unsatisfactory results were primarily due to under correction (10%) and poor lid crease (6%). Bhiromekraibhak et al. [17] described a technique using a double breast frontalis-orbicularis oculi muscle flap which the authors indicate enhances the mobility and amount of pretarsal orbicularis oculi muscle. Other authors have reported the use of silicone implant suspension in patients with poor or absent levator muscle function [18], [19]. Our results, including the 97.6% satisfied rate and low rate of complications compare very favorably with those reported by other studies [11], [12], [15]–[17].

When an incision under the eyebrow is used, it is relatively simple to create the frontalis muscle flap [20]. Local tissue trauma and swelling are minimal, and recovery is rapid. However, a scar will be left under the eyebrow although it is not obvious in most cases. When the incision is not made under the eyebrow, there is no scar but it is more difficult to create the frontalis muscle flap.

The mobility of tissue in children is far greater than that in adults, and we do not believe that the range of dissociation should be designed before surgery or the intraoperative dissociation should follow a predesigned range [21]. Appropriate downward traction of the frontalis muscle flap will not cause skin adhesions above the eyebrow or significant eyebrow displacement. The degree of the dissociation of the frontalis muscle flap and the skin should not be excessively great as this can result in an excessively long frontalis muscle flap. If the flap is too long the suspension force will be insufficient thus altering the desirable effects on eyelid motility.

The tunnel for the frontalis muscle flap can be created anterior or posterior to the orbital septum, based on the surgeon's experience and orientation. We prefer the tunnel be created posterior to the orbital septum, which is closer to the mechanism of the levator palpebrae superioris. During blunt dissection of the tunnel, the instruments should be passed through the fatty tissue; this allows the frontalis muscle flap to be wrapped in fat, which will minimize the occurrence of adhesions. The dissociation of the tunnel should be completed at one time in order to prevent the formation of multiple tunnels and cause excessive destruction of the fatty tissue. Even if the fatty tissue bulges, it should not be removed.

The upper eyelid skin is generally excessively long in patients with blepharoptosis, and should be resected appropriately in both bilateral blepharoptosis and unilateral blepharoptosis. The upper eyelid skin on the unaffected side should be treated with conventional double eyelid plasty in cases of unilateral blepharoptosis, though there is debate regarding this issue [22]. We typically remove a 1–3 mm wide strip of upper eyelid skin on the affected side.

The purpose of blepharoptosis surgery is to lift the drooping upper eyelid to a physiological level so that patients can look at objects naturally, and the height of the upper palpebral margin is critical [23]. Han et al. [24] thought that the upper eyelid on the affected side should be lifted to the level of the upper edge of the iris. Kim et al. [25] reported that postoperative eyelid height can be predicted by compensating for lagophthalmus due to anesthesia and in cases of more severe ptosis adjusting the palpebral fissure to be larger than the desired eyelid height. In our experience, the degree of lateral ptosis was more severe than that of the medial ptosis during the postoperative recovery process. A key point of this report is that we found that the height of the lateral fixation point should be 1 mm higher than that of the midpoint, making the highest point of the palpebral fissure at the junction between the medial and lateral 1/3 of the palpebral fissure. The appropriate height of suspension is to lift the midpoint of the affected upper palpebral margin until it is 1.5 mm or more above the upper edge of the iris. Using this technique a natural curvature of the palpebral margin in obtained in the long-term. It is important that when the frontalis muscle flap and the upper edge of the tarsus are sutured and fixed under general anesthesia, the orbital septum should not be sutured together in order to prevent palpebral fissure lagophthalmos. In addition, the fixation should be sturdy to prevent loosening and tearing. An additional stitch can be added between the fixation points.

In order to prevent the occurrence of complications and achieve the best surgical results, timely and comprehensive care is particularly important. Eye drops should be applied during the day, and eye ointment should be applied at night. The children should be told not to open or close the eyes forcibly in order to prevent frontalis muscle flap avulsion and aggravation of upper eyelid swelling. Of note, the technique we have described does not require the harvesting of autogenous fascia lata, which can be a source of postoperative morbidity [26]. It should also be noted that children of this age have difficulty cooperating for surgery, and are likely to move if local anesthetics are used; thus, the majority of the cases underwent general anesthesia.

## Conclusions

In summary, the results of this study indicate that the surgical correction of congenital blepharoptosis in early age children using a modification of frontalis muscle flap suspension provides satisfactory correction of blepharoptosis, natural and aesthetic double eyelid folds, and ideal eyelid position and curvature. For good surgical outcomes, attention should be given to preventing damage to the frontalis muscle flap during its preparation and adequate suspension height of the flap.

## Supporting Information

Table S1 Data of each case.
(DOC)
