# Supplementary material for: Frontalis Muscle Flap Suspension for the Correction of Congenital Blepharoptosis in Early Age Children
Source: PLoS One. 2013 Jan 7;8(1):e53185. doi: 10.1371/journal.pone.0053185 (PMC3538777; doi:10.1371/journal.pone.0053185)
Supplement: Table S1 — Data of each case. (DOC) [file pone.0053185.s001.doc]

**Table S**1. Data of each case

| **No** | **sex** | **Age, y** | **Anesthesia** | **Eye** | **Preoperative ptosis** | **Operation time, min** | **Blood loss, ml** | **Healing grade** | **Dysraphism at discharge, mm** | **Dysraphism at last follow-up, mm** | **Follow-up, mo** | **Complications** | **Patient satisfied** | **Reoperation for residual ptosis** |
| --- | --- | --- | --- | --- | --- | --- | --- | --- | --- | --- | --- | --- | --- | --- |
| 1 | girl | 6 | Local | Right | Moderate | 45 | 5 | Good | No | No | 6 | No | Yes | No |
| 2 | boy | 6 | General | Right | Moderate | 35 | 1 | Good | No | No | 6 | No | Yes | No |
| 3 | boy | 2.5 | General | Right | Moderate | 75 | 1 | Good | 1 | No | 3 | No | Yes | No |
| 4 | girl | 7 | Local | Right | Mild | 45 | 1 | Good | No | No | 12 | No | Yes | No |
| 5 | girl | 7 | General | Right | Severe | 35 | 1 | Good | < 0.5 | No | 3 | No | Yes | No |
| 6 | girl | 4.5 | General | Right | Severe | 45 | 1 | Good | No | No | 3 | No | Yes | No |
| 7 | boy | 7 | General | Right | Severe | 60 | 3 | Good | No | No | 12 | No | Yes | No |
| 8 | girl | 4 | General | Right | Severe | 40 | 1 | Good | No | No | 3 | No | Yes | No |
| 9 | boy | 7 | General | Right | Moderate | 45 | 10 | Good | No | No | 3 | No | Yes | No |
| 10 | boy | 4 | General | Right | Severe | 45 | 1 | Good | 5 | 5 | 12 | Dysraphism | Yes | No |
| 11 | boy | 3 | General | Right | Severe | 35 | 3 | Good | No | No | 6 | No | Yes | No |
| 12 | boy | 6 | General | Right | Moderate | 50 | 20 | Good | No | No | 6 | No | Yes | No |
| 13 | boy | 9 | General | Right | Moderate | 50 | 1 | Good | No | No | 3 | No | Yes | No |
| 14 | boy | 3 | General | Left | Severe | 85 | 1 | Good | No | No | 12 | No | Yes | No |
| 15 | boy | 6 | General | Left | Severe | 35 | 1 | Good | No | No | 3 | No | Yes | No |
| 16 | boy | 6 | Local | Left | Mild | 65 | 1 | Good | No | No | 3 | No | Yes | No |
| 17 | boy | 8 | General | Left | Mild | 60 | 1 | Good | < 0.5 | No | 3 | No | Yes | No |
| 18 | girl | 4 | General | Left | Severe | 35 | 1 | Good | No | No | 12 | No | Yes | No |
| 19 | boy | 8 | General | Left | Moderate | 50 | 1 | Good | No | No | 6 | No | Yes | No |
| 20 | boy | 5 | General | Left | Mild | 55 | 1 | Good | No | No | 3 | No | Yes | No |
| 21 | girl | 5.5 | General | Left | Severe | 85 | 1 | Good | No | No | 6 | No | Yes | No |
| 22 | girl | 7 | Local | Left | Severe | 60 | 1 | Good | 3 | 3 | 3 | Dysraphism | Yes | No |
| 23 | boy | 6 | General | Left | Severe | 50 | 1 | Good | No | No | 3 | No | Yes | No |
| 24 | boy | 4 | General | Left | Severe | 55 | 1 | Good | No | No | 12 | No | Yes | No |
| 25 | boy | 6 | General | Left | Severe | 105 | 1 | Good | No | No | 3 | No | Yes | No |
| 26 | girl | 8 | General | Left | Severe | 55 | 10 | Good | No | No | 12 | No | Yes | No |
| 27 | boy | 9 | General | Left | Moderate | 45 | 1 | Good | < 0.5 | No | 12 | No | Yes | No |
| 28 | boy | 9 | General | Left | Severe | 70 | 1 | Good | 1 | 1 | 12 | Dysraphism | Yes | No |
| 29 | girl | 5 | Local | Left | Severe | 55 | 1 | Good | No | No | 12 | No | Yes | No |
| 30 | boy | 5 | General | Left | Moderate | 50 | 20 | Good | No | No | 3 | No | Yes | No |
| 31 | boy | 6 | General | Left | Severe | 140 | 1 | Good | No | No | 12 | No | Yes | No |
| 32 | girl | 9 | Local | Left | Severe | 60 | 1 | Good | No | No | 3 | No | Yes | No |
| 33 | girl | 8 | General | Left | Severe | 45 | 1 | Good | No | No | 3 | No | Yes | No |
| 34 | boy | 6 | General | Left | Moderate | 60 | 1 | Good | No | No | 3 | No | Yes | No |
| 35 | boy | 9 | Local | Left | Moderate | 60 | 1 | Good | No | No | 12 | No | Yes | No |
| 36 | boy | 7 | General | Bilateral | Moderate | 35 | 1 | Good | No | No | 12 | No | Yes | No |
| 37 | boy | 3 | General | Bilateral | Moderate | 80 | 1 | Good | No | No | 6 | No | Yes | No |
| 38 | boy | 8 | Local | Bilateral | Moderate | 125 | 10 | Good | No | No | 12 | No | Yes | No |
| 39 | girl | 8 | General | Bilateral | Severe | 75 | 1 | Fair | 2 | 2 | 12 | Dysraphism | Yes | No |
| 40 | boy | 6 | General | Bilateral | Severe | 65 | 1 | Fair | No | No | 3 | No | Yes | No |
| 41 | girl | 6 | General | Bilateral | Severe | 65 | 1 | Good | No | No | 12 | No | Yes | No |
| 42 | girl | 7 | General | Bilateral | Severe | 45 | 1 | Good | No | No | 6 | No | Yes | No |
| 43 | boy | 4 | General | Bilateral | Mild | 60 | 1 | Good | No | No | 12 | No | Yes | No |
| 44 | boy | 5 | General | Bilateral | Severe | 60 | 1 | Good | No | No | 3 | No | Yes | No |
| 45 | boy | 8 | Local | Bilateral | Moderate | 45 | 90 | Good | No | No | 3 | No | Yes | No |
| 46 | boy | 6 | General | Bilateral | Severe | 45 | 5 | Good | No | No | 6 | No | Yes | No |
| 47 | boy | 10 | General | Bilateral | Moderate | 65 | 1 | Good | No | No | 3 | No | Yes | No |
| 48 | girl | 7 | General | Bilateral | Severe | 75 | 1 | Good | No | No | 12 | No | Yes | No |
| 49 | girl | 8 | General | Bilateral | Severe | 80 | 20 | Good | No | No | 6 | No | Yes | No |
| 50 | boy | 8 | General | Bilateral | Mild | 80 | 1 | Good | No | No | 60 | No | Yes | No |
| 51 | boy | 7 | General | Bilateral | Severe | 110 | 1 | Good | No | No | 12 | No | Yes | No |
| 52 | boy | 4 | General | Bilateral | Severe | 90 | 5 | Good | No | No | 3 | No | Yes | No |
| 53 | girl | 5.5 | General | Bilateral | Severe | 90 | 20 | Good | No | No | 6 | No | Yes | No |
| 54 | girl | 3 | General | Right | Severe | 55 | 1 | Good | No | No | 6 | No | Yes | Yes |
| 55 | boy | 7 | General | Right | Moderate | 35 | 1 | Good | No | No | 12 | No | Yes | Yes |
| 56 | boy | 8 | General | Right | Moderate | 35 | 1 | Good | No | No | 12 | No | Yes | Yes |
| 57* | boy | 4 | General | Bilateral | Severe | 55 | 1 | Good | No | No | 12 | No | Yes | Yes |
| 58* | boy | 4 | General | Bilateral | Severe | 60 | 1 | Good | No | No | 6 | No | Yes | Yes |
| 59* | boy | 5 | General | Bilateral | Severe | 65 | 1 | Good | No | No | 12 | No | Yes | Yes |
| 60 | girl | 3 | General | Bilateral | Severe | 70 | 1 | Good | No | No | 6 | No | Yes | Yes |
| 61 | boy | 5 | General | Right | Severe | 60 | 1 | Good | 1 | 1 | 3 | Dysraphism | Yes | No |

All patients had congenital blepharoptosis and a negative family history of ptosis. Frontalis muscle flap suspension was performed in all patients.

*Indicates patient also had microphthalmia.
